# The eyes test is influenced more by artistic inclination and less by sex

**DOI:** 10.3389/fnhum.2015.00292

**Published:** 2015-05-22

**Authors:** Paola Guariglia, Laura Piccardi, Flavio Giaimo, Sofia Alaimo, Giusy Miccichè, Gabriella Antonucci

**Affiliations:** ^1^Dipartimento Scienze dell’Uomo e della Società, Università degli Studi KoreEnna, Italy; ^2^Dipartimento di Medicina Clinica, Sanità Pubblica, Scienza della Vita e dell’Ambiente, Università degli Studi dell’AquilaL’Aquila, Italy; ^3^Unità di Neuropsicologia, IRCCS Fondazione Santa LuciaRome, Italy; ^4^Dipartimento di Psicologia, Università degli Studi di Roma “La Sapienza”Rome, Italy

**Keywords:** RMET, empathy, sex differences, artistic attitude, emotions

## Abstract

The “Reading the Mind in the Eyes” test was developed by Baron-Cohen and his co-workers. This test provides them the unique opportunity to evaluate social cognition assessing the ability to recognize the mental state of others using only the expressions around the eyes. In healthy populations, however, it has produced conflicting results, particularly regarding sex differences and number of items to use. In this study we performed two studies: The first one investigated the presence of gender effects and the sensitivity of test stimuli; the second one considered other individual factors (i.e., artistic attitude, social empathy and personality traits) that could influence the ability to understand emotions from gaze. Our results demonstrated a sex effect, which can be more or less attenuated by the nature of the stimuli. This could be as aforementioned the result of the following, empathy or artistic attitude in being proficient in understanding the mental states of others.

In the general population, there are individual differences in prosocial behavior. Specifically, social emotions function as emotional responses to unfair or fair decisions and social reasoning assesses how others are likely to act in a given situation. Empathy is considered to have a crucial role in social interaction, since it allows sharing the social emotions of others (Singer, 2008) contributing to provide crucial information for the adaptation to the world promoting communication and social relationships (Ekman et al., 1987; Ekman, 2009).

Emotion recognition is the ability to read subtle cues (i.e., facial expression, prosody) that indicate others’ emotional states (Gallese et al., 2004, 2009; Adolphs, 2009). Human faces provide several emotional cues, but it is difficult to disentangle their real emotional meaning (Bartlett et al., 1999). When observing a face, humans orient their attention towards some core facial features (van der Geest et al., 2002). The eye and mouth regions not only provide information about a person’s identity, but also about their mental state. From a developmental point of view, from about 10 weeks of age the internal elements are more fixed than the external ones, with 90% of fixations directed towards inner elements (i.e., eyes and mouth; Hunnius and Geuze, 2004). This is in line with the Baron-Cohen’s (Baron-Cohen, 1997) hypothesis that interprets gaze as crucial in reading other people’s thoughts and intentions. Baron-Cohen and his colleagues (Baron-Cohen et al., 1997a,b, 2001) developed the “Reading the Mind in the Eyes” test (RMET), for evaluating social cognition by assessing the ability to recognize mental states of others just by the expression around their eyes. This test has been largely used in experimental, neuroimaging and clinical studies to detect differences in emotional attribution due to eye reading in both clinical populations (i.e., in patients with schizophrenia, autism, eating disorders and social anxiety) and in healthy participants (i.e., to investigate sex-related and age-related changes) (Vellante et al., 2013).

Sex differences in recognizing emotions are still a topic of debate. Specifically, a meta-analysis reported that 80% of studies show women advantage (Hall et al., 2000; Carroll and Chiew, 2006; Hoffmann et al., 2010), with relatively small effect sizes, other studies did not find these differences (Grimshaw et al., 2004; Kessler et al., 2005) and other studies report gender effects favoring men (e.g., Nettle and Liddle, 2008). In a more recent meta-analysis, Kirkland et al. (2013) found only a small mean effect size in favor of women over men on the RMET.

There are several reasons why we should expect to find sex differences in emotional recognition by reading facial expression. First, sex differences emerged in girl infants’ attention to faces, that attend more to a face than boy infants, who attend more to moving objects (Connellan et al., 2000). Very early sex differences in eye-to-eye contact have also been described, with a double effect, on the quality of social behaviors and on exposure to faces (Hittelman and Dickes, 1979). Further, there are reports that in early and late infancy, childhood and adulthood females tend to engage in mutual eye contact or focus on the eyes more than males (Exline et al., 1965; Ashear and Snortum, 1971; Levine and Sutton-Smith, 1973; Osofsky and O’Connel, 1977; Field et al., 1984; Hall et al., 2010; Saether et al., 2009). On the basis of this kind of precocious behavior, women should recognize, discriminate between, and interpret facial expressions better than men (Hall, 1978; McClure, 2000).

Other individual differences are related to personality traits that drive people in choosing a profession. Specifically, a study found that surgeons are less empathetic than psychiatrists (Dehning et al., 2013). Moreover, another study found that higher levels of empathy drive the choice of first-year medical students and their preference for a specialty with continuous patient care (Dehning et al., 2014). Wheelwright et al. (2006) also showed that cognitive styles could be related to professional choice. In particular, they observed that people with a systemizing cognitive style (i.e., with high ability to analyse the rules underlying a system, in order to predict its behavior) tend toward science and mathematics, differently from people with an empathizing style (i.e., with high ability to identify another’s mental states and to respond to these with one of a range of appropriate emotions). In line with this result, Billington et al. (2007) have observed that systemizing and empathizing cognitive styles individuals perform on RMET in a different way and that empathizing people are better than the others.

Both Wheelwright et al. (2006) and Billington et al. (2007) adopted the RMET and empathy scales to assess their hypothesis. Altogether these studies suggest that personal attitude towards a profession might be related to level of empathy and to the ability to read social emotions. The professional choice to cure diseases implies in any case an interest toward the suffering of the others and it is possible that also the less empathic surgeons are indeed more empathic than other type of professionals. Indeed, it is generally accepted that people who choose professions aimed to cure others (physicians, nurses, teachers, etc.) have more empathic attitude than others. We wondered if also another type of professionals, who express others’ feelings without taking care of others, namely artistic professionals, show better performances on RMET suggesting better empathic capabilities. However, to our knowledge, no studies have investigated whether artists are more empathic than non artists and we can hypothesize that as in the care-taking professions, an artist need to interpret and to represent self and others emotions.

The aims of this study were twofold: First, we wanted to confirm the presence of sex differences in solving the RMET. Second, we wanted to investigate whether or not the artists perform better on the RMET than non artists and if this ability is also related to empathy levels. Thus, we planned two studies, one investigating sex differences and the other artistic competence, the influence of degree of empathy and personality traits.

## Experiment 1

We investigated the presence of gender effects and the nature of the RMET’s items that evidenced these differences.

### Method

#### Participants

One-hundred forty college students (70 men), aged from 20–30 years (men: *M* = 24.96, S.D. = 3.52; women: *M* = 24.54, S.D. = 3.39), without neurological or psychiatric disorders were enrolled in the study. They gave their written informed consent to participate and were tested according to the guidelines of the local ethics committee, which were in line with the principles of the Declaration of Helsinki.

#### Instruments and Procedure

*RMET* (Baron-Cohen et al., 2001; Vellante et al., 2013).

**Figure 1:**
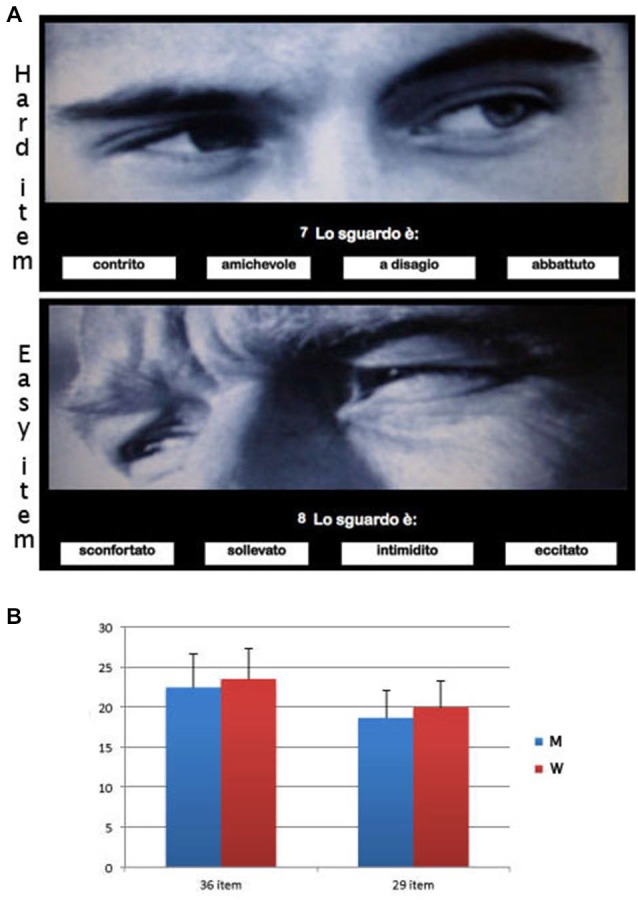
**(A)** Examples of eliminated items on the basis of results of the Item Difficulty Analysis: Hard item (IDA = 0, 105; “a disagio” (uncomfortable) is the target, or correct answer); Easy item (IDA = 0, 829; “sconfortato” (disconsolate) is the target, or correct answer). **(B)** Means and standard deviations of men and women groups in the *Reading the Mind in the RMET* (standard version: 36 items vs. shortened version: 29 items).

Participants were randomly presented with a series of 36 photographs of the eye region of 19 actors and 17 actresses. Each photo was surrounded by four single-word mental state descriptors (e.g., bored, angry). One of these descriptors targeted the mental state depicted in the photo, and the others were foils (Figure 1). The RMET is based on a four-alternative forced-choice paradigm, with 25% correct guess rate. Participants had to choose which of the four descriptors best described what the person in the photo was thinking or feeling. If participants were unsure of the definition they could also ask the examiner to explain the meaning of the descriptors. The test score was the number of descriptors correctly identified. The maximum score was 36. Participants could take only one minute per item before they had to move on to the next item. They were tested individually in a quiet room with artificial lighting and seated on a height-adjustable chair in front of computer screen on which the RMET was shown. The examiner, who was behind them, recorded their answers on the answer sheet. Participants gave a verbal response for the state descriptor identified.

### Results

A one-way ANOVA was carried out with Group (M and F) as independent variable and corrected answer (hits) in solving the RMET (*F*_(1,138)_ = 2.17; *p* = 0.14). As some authors have explained the inconsistency across studies as due to differences in the nature of the stimuli (Hoffmann et al., 2010), we decided to perform a Quantitative Item Analysis, specifically, an Item Difficulty Analysis (IDA) to detect items that could be removed because they were too easy or too difficult. The item difficulty index can be computed by simply dividing the number of test takers who answered the item correctly by the total number of participants who answered the item. As a proportion, this index can range between 0.00, obtained when no participants answered the item correctly, and 1.00, obtained when all participants answered the item correctly. We excluded one item with a difficulty level of 0.20 (20% of the participants answered the item correctly) and of 0.80 (80% of the participants failed to answer the item). On the basis of results of the IDA we eliminated seven items (i.e., items 7, 8, 18, 19, 23, 25, and 31; specifically, items 8 and 18 were >0.08 and the others were ≤0.02) (see Figure 1A, for examples of easy and difficult items).

After performing the IDA we performed a one-way ANOVA with Group (M and F) as independent variable and corrected answer (hits) in solving 29-items RMET (*F*_(1,138)_ = 5.02; *p* = 0.027; partial eta-squared = 0.29) in which women performed better than men in attributing the correct state descriptors to the picture (Figure 1B).

## Experiment 2

We investigated whether artistic aptitude makes individuals more prone to detecting emotions from others’ facial expressions and whether this aptitude is related to higher empathy scores. We also assessed personality traits (i.e., extroversion vs. introversion) hypothesizing that artists could be more extroverted and as a consequence more proficient in understanding social emotions.

### Participants

One-hundred participants (50 artists (A) and 50 non-artists (NA); A: *M* = 28.80, S.D. = 13.60 years of age; NA: *M* = 31.30, S.D. = 15.19 years of age) participated in the study. No participants had a history of neurological and/or psychiatric disorders.

The artist group (9 painters; 5 sculptors; 24 musicians and 12 dancers) was recruited through advertisements posted at the School of Fine Arts, the Conservatory and the Ballet Music Schools in the geographic areas of Caltanissetta and Palermo (Sicily, Italy). We assessed both teachers and students at the end of their educational program. The non-artist group had no artistic training and was comparable with A group for age, sex and educational level.

All participants gave their written informed consent and were tested according to the guidelines of the local ethics committee, which were in line with the principles of the Declaration of Helsinki.

### Instruments and Procedure

All participants were administered the *RMET* (Baron-Cohen et al., 2001; Vellante et al., 2013) following the same procedure as in Experiment 1; they were also administered several scales and a questionnaire to determine their empathy level and personality dimensions. We decide to investigate these aspects since the RMET performance seems to be sensitive to several variables (i.e., General Intelligence; Cognitive Style; Sex; Empathy).

Eysenck Personality Questionnaire-Revised (EPQ-R; Eysenck et al., 1985; Dazzi et al., 2010) is a self-reported questionnaire with 48 items (12 for each of the traits of neuroticism, extraversion, and psychoticism, and 12 for the lie scale). Each question has a “yes” or “no” response scored 1 or 0 and each scale has a maximum possible score of 12. Specifically, the traits measured belonged to the following personality dimensions:

1. Neuroticism: People with high N scores tend to be emotionally over responsive and have difficulty calming down. They often complain of vague somatic upsets and report preoccupation, anxiety and irritated emotional feelings;
2. Extraversion-Introversion: Individuals with a high E score tend to be outgoing, impulsive, uninhibited, have many social contacts and often take part in to group activities. Typically, the extravert is highly social, likes gatherings, has many friends, needs to have people to talk to and dislikes solitary pursuits such as reading, studying, and contemplation;
3. Psychoticism: People with high P scores are inclined to be cruel, inhumane, socially indifferent, hostile, aggressive and oblivious to danger, insular, glacial, intolerant and lacking in empathy;
4. Lie scale: considers behaviors that are either socially desirable but infrequently practised or frequently practised but socially undesirable to detect any false answers given in the other three scales.

Empathy Quotient (EQ; Baron-Cohen and Wheelwright, 2004). This is a 60-item questionnaire designed to measure empathy in adults. Each item is a first person statement, which the participant must rate as Strongly Agree, Slightly Agree, Slightly Disagree or Strongly Disagree. The instrument is scored on a scale of 0 (the least empathetic possible) to 80 (the most empathetic possible). A useful cut-off of 30 was established to screen for Autism Spectrum Disorders (Baron-Cohen and Wheelwright, 2004).

Participants were tested individually in a quiet room with artificial lighting. They performed the RMET and the other scales. The administration order of the RMET and the other scales was counterbalanced across subjects.

### Results

**Figure 2:**
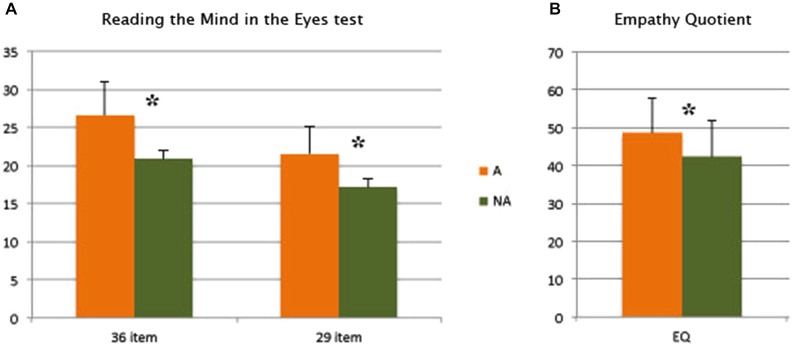
**Means and standard deviations of Artist and No-Artist groups: (A) in the *Reading the Mind in the RMET* (standard version: 36 items and shortened version: 29 items); (B) in the *Empathy Quotient***.

Figure 2A illustrates the means and standard deviations of the correct answers provided by Artist and Non-artist groups in performing the RMET in both the standard version (36 items) and the shortened version (29 items), depending on the results of Experiment 1; Figure 2B shows means and standard deviations of A’s and NA’s performance in the EQ test.

Two one-way ANOVA with Group (A and NA) as independent variable and corrected answers (hits) in solving the 36-item or the 29-item RMET showed a significant difference (*F*_(1,98)_ = 43.09; *p* < 0.001 and *F*_(1,98)_ = 36.75; *p* < 0.001) between A and NA. In both analyses, the artists were more successful than the non-artists in attributing the correct state descriptors to the picture (Figure 2A).

We also performed separate one-way ANOVAs for the scores obtained by A and NA in the EPQ-R (the 4 subscales)and EQ.

The one-way ANOVA for EPQ-R showed no significant differences (Neuroticism: *F*_(1,98)_ = 0.11; *p* = 0.74; Extroversion-Introversion: *F*_(1,98)_ = 2.15; *p* = 0.15; Psychoticism: *F*_(1,98)_ = 2.87; *p* = 0.09; Lie: *F*_(1,98)_ = 0.19; *p* = 0.67) between A and NA. Differently, the one-way ANOVA performed for EQ evidenced a significant difference (*F*_(1,98)_ = 14.23; *p* = 0.0003) for A and NA (Figure 2B), specifically the A group was more empathetic than the NA group.

We also performed Pearson’s correlation on the tests (the RMET, the EQ and the subscales of the EPQ-R) and found that only the EQ results correlated with those of the RMET. The other subscales were correlated with each other for several different measures but not the RMET (see Table 1 for details).

**Table 1:** **Pearson’s correlation on all tests**.

	E	N	P	L	EQ	RMET
E	1	−0.261**	0.052	−0.026	0.241*	0.162
N	−0.261**	1	0.105	−0.109	−0.038	−0.094
P	0.052	0.105	1	−175	0.103	0.038
L	−0.026	−0.109	−0.175	1	0.254**	−0.062
EQ	0.241*	−0.038	0.103	0.254**	1	0.204*
RMET	0.162	−0.094	0.038	−0.062	0.204*	1

**p < 0.01; *p < 0.05. Tests: E = Extraversion-Introversion by EPQ-R; N = Neuroticism by EPQ-R; P = Psychoticism by EPQ-R; L = Lie scale by EPQ-R; EQ = Empathy Quotient and RMET.

As there was a correlation between the EQ and the RMET we performed separate one-way ANOVAs for each group taking into account sex. Regarding the A group, we observed no significant difference between men and women in performing the short (*F*_(1,48)_ = 3.40; *p* = 0.07) and long version (*F*_(1,48)_ = 2.70; *p* = 0.11) of the RMET, but women were significantly more empathetic than men in the EQ (*F*_(1,48)_ = 4.61; *p* = 0.04). The NA group performances on both the short (*F*_(1,48)_ = 0.15; *p* = 0.70) and long versions (*F*_(1,48)_ = 0.17; *p* = 0.68) of the RMET and EQ (*F*_(1,48)_ = 2.83; *p* = 0.10) did not differ between men and women.

## Discussion

In the present study our aims were twofold: (1) to confirm the presence of effects, favoring women, on the RMET; and (2) to determine the role of artistic attitude, empathy level and personality traits in performance on the RMET.

With respect to the first aim, we found, as largely demonstrated in literature (see the meta-analysis by Kirkland et al., 2013), an advantage of women in performing shortened RMET. This result is in line with the reports in the literature indicate that women’s social behavior is precocious, which should explain their better ability to recognize, discriminate between and interpret facial expressions, we assessed two large groups of college students by asking them to perform the RMET (Baron-Cohen et al., 2001). This test is widely used in both experimental and clinical settings to identify an individual’s emotional state. Indeed, it was originally developed to study high-functioning individuals with autism (Baron-Cohen and Hammer, 1997; Baron-Cohen et al., 1997a,b) but has also proved valuable for investigating individual differences among normally developing samples (Baron-Cohen et al., 2001). In 2001, Baron-Cohen and colleagues developed a revised version of the RMET, which has more items than the previous test (36 vs. 25 items) and more emotional descriptors (two vs. four descriptors) for each item.

Vellante et al. (2013) reported that in 6 out of 17 studies a statistically significant women advantage was observed, with Cohen’s d ranging from 0.22–0.94. However, our data show gender differences only in the shortened 29-item version of RMET; indeed, when we used the revised version of the RMET (Baron-Cohen et al., 2001), no gender differences emerged.

Peterson and Miller (2012) reported that in a test-retest reliability study 7 items reduced the overall alpha; therefore, they administered the RMET with 29 items without specifying which of the 36 original items were included. Also other studies (Serafin and Surian, 2004; Voracek and Dressler, 2006; Harkness et al., 2010; Dehning et al., 2012) found low internal coherence measured by Cronbach’ s alpha or Guttman’ s split-half methods. In a meta-analysis including more than 4000 subjects Kirkland et al. (2013) found “*A small but significant effect in favor of females*” and discussed that the effect size is most likely an underestimation due to the reliability of the RMET. The item analysis performed in present study support Kirkland et al.’s observation about the reliability of the RMET, also suggesting that effect they found in their meta-analysis would result significantly greater if the seven items we identified as less sensitivity could be excluded. Further, several variables seem to affect the performance on the RMET, for instance Baker et al. (2014) in another meta-analysis found that performance on the RMET positively correlates with intelligence without a specific correlation with performance or verbal ability.

Our finding is in line both with Peterson and Miller (2012) and Kirkland et al. (2013) findings and leaves open the questions about the structure and item sensitivity of the RMET. In any case, we retain that present results provide useful information about the sensitivity of the single items for all the future studies (for example, fMRI studies) in which, due to methodological issues, the full RMET could not be presented and researches have to select some items to be presented.

The results of our experiment 1 confirm the variability in detecting stable gender effects and suggest that other individual differences might be important in understanding social emotions. For this reason, we planned a second experiment in which we assessed other aspects that might be important in determining proficiency in understanding social emotions. Similar to the studies on empathy performed by (Dehning et al., 2013, 2014), in which the authors took into account professional choice hypothesizing that surgeons are less empathetic than psychiatrists (Dehning et al., 2014), we considered artistic attitude. We hypothesized that artists would be more empathetic and also more able to understand social emotions than non artists of the same age and the same level of education. Our results confirmed the greater proficiency of artists in solving the RMET and also a general higher empathy level in this group with respect to the non-artists. We also considered personality traits in the two groups. Although several aspects of personality correlated with each other, they did not correlate with the RMET performance. Differently, empathy level was the only one that correlated with the RMET, suggesting its crucial role in social emotions. Also in Vellante et al. (2013) those participants who scored lower on the EQ also scored lower on the RMET than those who did not. This data emerge also in earlier reports of females scoring higher than males on tests of empathy (Eisenberg and Lennon, 1983; Baron-Cohen and Wheelwright, 2004; Lawrence et al., 2004; Von Horn et al., 2010).

We can speculate that the gender effects observed in solving the RMET are due to higher empathy levels in women than in men. Indeed, previous studies found that women were better at recognizing and sharing others’ emotions (Luo et al., 2014; Van der Graaff et al., 2014). Moreover, women showed also more often than men an empathizing style as reported by Billington et al. (2007), therefore it becomes difficult to disentangle the role of sex and the role of a higher level of empathy.

If empathy is the key factor that leads to gender differences, it is possible that the inconsistent results in the literature are due to the different level of empathy in women and men rather than different capacities to solve the RMET.

The present study suggests that the RMET should be used together with other tools (i.e., empathy scales) to disentangle the role of empathy in attributing emotions.

In conclusion, the RMET is highly sensitive in discriminating performances in pathological samples and for this reason, it is widely used in clinical settings. However, when used to detect slight differences in healthy people it is difficult to understand what determines better performance; indeed, several variables (i.e., gender, empathy, artistic attitudes, professional choices, etc.) seem to affect performance. In general, with healthy participants the short 29-item version seems to be more sensitive than the 36-item version. Further studies with large, balanced samples of men and women in which empathy levels, RMET results and other variables are considered could be useful to understand the potential application of this interesting tool.

## Conflict of Interest Statement

The authors declare that the research was conducted in the absence of any commercial or financial relationships that could be construed as a potential conflict of interest.
